# Diversity among African Pygmies

**DOI:** 10.1371/journal.pone.0013620

**Published:** 2010-10-26

**Authors:** Fernando V. Ramírez Rozzi, Marina L. Sardi

**Affiliations:** 1 UPR 2147 CNRS, Paris, France; 2 División Antropología, Facultad de Ciencias Naturales y Museo, Universidad Nacional de La Plata, La Plata, Argentina; State University of New York College at Oneonta, United States of America

## Abstract

Although dissimilarities in cranial and post-cranial morphology among African pygmies groups have been recognized, comparative studies on skull morphology usually pull all pygmies together assuming that morphological characters are similar among them and different with respect to other populations. The main aim of this study is to compare cranial morphology between African pygmies and non-pygmies populations from Equatorial Africa derived from both the Eastern and the Western regions in order to test if the greatest morphological difference is obtained in the comparison between pygmies and non-pygmies. Thirty three-dimensional (3D) landmarks registered with Microscribe in four cranial samples (Western and Eastern pygmies and non-pygmies) were obtained. Multivariate analysis (generalized Procrustes analysis, Mahalanobis distances, multivariate regression) and complementary dimensions of size were evaluated with ANOVA and post hoc LSD. Results suggest that important cranial shape differentiation does occur between pygmies and non-pygmies but also between Eastern and Western populations and that size changes and allometries do not affect similarly Eastern and Western pygmies. Therefore, our findings raise serious doubt about the fact to consider African pygmies as a homogenous group in studies on skull morphology. Differences in cranial morphology among pygmies would suggest differentiation after divergence. Although not directly related to skull differentiation, the diversity among pygmies would probably suggest that the process responsible for reduced stature occurred after the split of the ancestors of modern Eastern and Western pygmies.

## Introduction

The term ‘pygmy’ regroups all human populations with average adult size lower than 155 cm [1]. Many African human populations show a short adult size with an average stature ranking between 155 and 160 cm. These populations sometimes called ‘pygmoid’ show a large geographical distribution and inhabit diverse and contrasted environments. African pygmies live in the equatorial rainforest and their phenotype has been explained by an adaptation to the rainforest habitat [1]–[5], [see 6 for a recent review]. They share a semi-nomadic way of life with a forager strategy and are associated with farming societies [7]. Many works have researched the genetic and/or endocrinological basis of the small size in Pygmies; however the reason of the reduced size remains at moment elusive (see below).

African pygmies can be divided into two main groups: Western pygmies inhabiting Cameroon, Central-African Rep., Gabon and Congo and Eastern pygmies located at the Nord-East of the Democratic Rep. of Congo and Rwanda. The hypothesis of a common origin for all worldwide pygmies' [8]–[9] has long time ago been abandoned. Although a different origin for East and Western pygmies has been suggested by Hiernaux [10], [11] and Cavalli-Sforza et al. [12] the idea of a common origin for African pygmies persists [e.g. 13]–[15]. A recent genetic study has suggested that African pygmies have diverged from the common ancestor with other African groups around 60–70,000 years ago [13]–[16] and that Eastern and Western pygmies diverged around 20,000 years ago [14]–[15].

Sub-Saharan Africa is the earliest region inhabited by modern humans and presents the greatest biological variation between native populations according with several biological indicators [17]–[20]. The history of this region has been marked by the Bantu expansion which occurred 3,000 yrs BP. Although the origin, routes of migrations and process of population dispersions as well as technological diffusion are still debated, the Bantu expansion produced a homogenization of physical traits reducing regional differences. However, cranial morphology seems to express an important differentiation [17]. Although pygmies adopted the language of their non-pygmies neighbours (with the exception of Baka), the admixture between pygmies and non-pygmies individuals is not frequent probably due to cultural barriers [16], [21]. It is supposed that Western pygmies present a higher level of admixture than Eastern pygmies explaining the taller stature of the former than the latter. The degree of admixture varies in a same region, e.g. Kola pygmies from South-west Cameroon show a higher level of admixture than Baka pygmies from South-east Cameroon [13]. Despite this fact, genetics studies indicate a quite clear distinction between pygmies and non-pygmies [13]–[16]. Thus, African pygmies appear as an assemblage of distinctive populations [22]–[24].

This distinction often leads to some scholars to pool all pygmies together in comparative studies of skull morphology [e.g. 17], [21], [25], [26], however some of them remarked morphological differences [18]. Marquer [27] and Vallois and Marquer [28] carried out a descriptive morphological study in Eastern and Western Pygmies and compared them with non-pygmies groups living in the same environment and sharing similar subsistence strategy. Although less marked than post-cranial ones, differences in skull morphologies have been observed among pygmies [27]. Twiesselmann [29], based on a comparative craniofacial work, suggested that differences between pygmies and non-pygmies from West Africa are less important than those between Eastern and Western pygmies. Froment [21] and Marquer [27] fail to find any craniological trait common to all pygmies and their distinction with respect to non-pygmy skulls remains difficult to do with confidence. It seems that behind the fact of pooling all pygmies together underlies the assumption that because they share the reduced stature and a similar way of life, all morphological characters are similar among them and very different with respect to other populations. Furthermore, studies based on mtDNA observed a great distance between Eastern and Western Pygmies [15]–[16] and recently Patin et al. [14], based on the study of the sequence variation of DNA samples, suggested that non-pygmy populations, Eastern pygmies and Western pygmies can be broadly classified in three different genetic entities.

It is not known, on the other hand, if cranial morphology would be associated with adult size in modern human populations. Allometries in African pygmies were tackled by Shea and Gomez [30] and Shea and Bailey [31]. Based on the comparison of body, tooth and skull dimensions in Asian and African pygmies and non-pygmies populations, Shea and Gomez [30] concluded that tooth dimensions in pygmies are not different to those in non-pygmies groups and suggest that the lack of allometry between body size and tooth size can be due to the early development of teeth. In a posterior work, Shea and Bailey [31] evaluated body proportions in order to test the hypothesis that morphologic differentiation of African pygmies can be explained by the differential extension of a common growth pattern (i.e. ontogenetic scaling). They observed that particular body proportions reported in pygmies result by a “nonadaptive allometric correlates of overall size reduction” [31: 331] and suggest a truncation of growth as the explanatory mechanism of pygmy morphology.

The biological mechanisms that produced the reduced adult stature in African pygmies remain elusive. The most accepted hypothesis, for explaining this phenotype, suggests that the short stature of adult Pygmies is due to some kind of deficiency in the growth hormone – insuline-like growth factor 1 axis (GH-IGF1) [e.g.32]–[42]. It is not clear if few genes are responsible of the size effect (e.g., GH, GH-BP, IGF1) or if the reduced size results from the accumulation of small mutations implicating several genes. Results are contradictory and since no underlying molecular defect has been identified, all suggestions remain inconclusive [43].

This work presents several aims oriented to test if African pygmies can be considered as before a unique group when compared with other populations. The main aim of this study is to compare cranial morphology between African pygmies and non-pygmies populations from Equatorial Africa derived from both the Eastern and the Western regions. Since the pattern of cranial differentiation reflects a great proportion of population history and considering previous studies, the **first hypothesis** to be tested is that pygmy populations form an homogenous group with respect to non-pygmies, thus we expect that the greatest morphological difference is obtained in the comparison between pygmies' and non-pygmies'.

Since cranial morphology would be associated with postcranial morphology, other hypotheses have been tested in order to get more insight on biological mechanisms of morphologic differentiation. The **second hypothesis** states that pygmies show smaller craniofacial size than non-pygmies populations because cranial size may be associated with body size. Since cranial size differentiation may be associated with shape variation (allometries), the **third hypothesis** proposes that particular cranial shape between pygmies and non-pygmies is associated with size differentiation.

## Results

### Cranial shape and size

**Figure 1:**
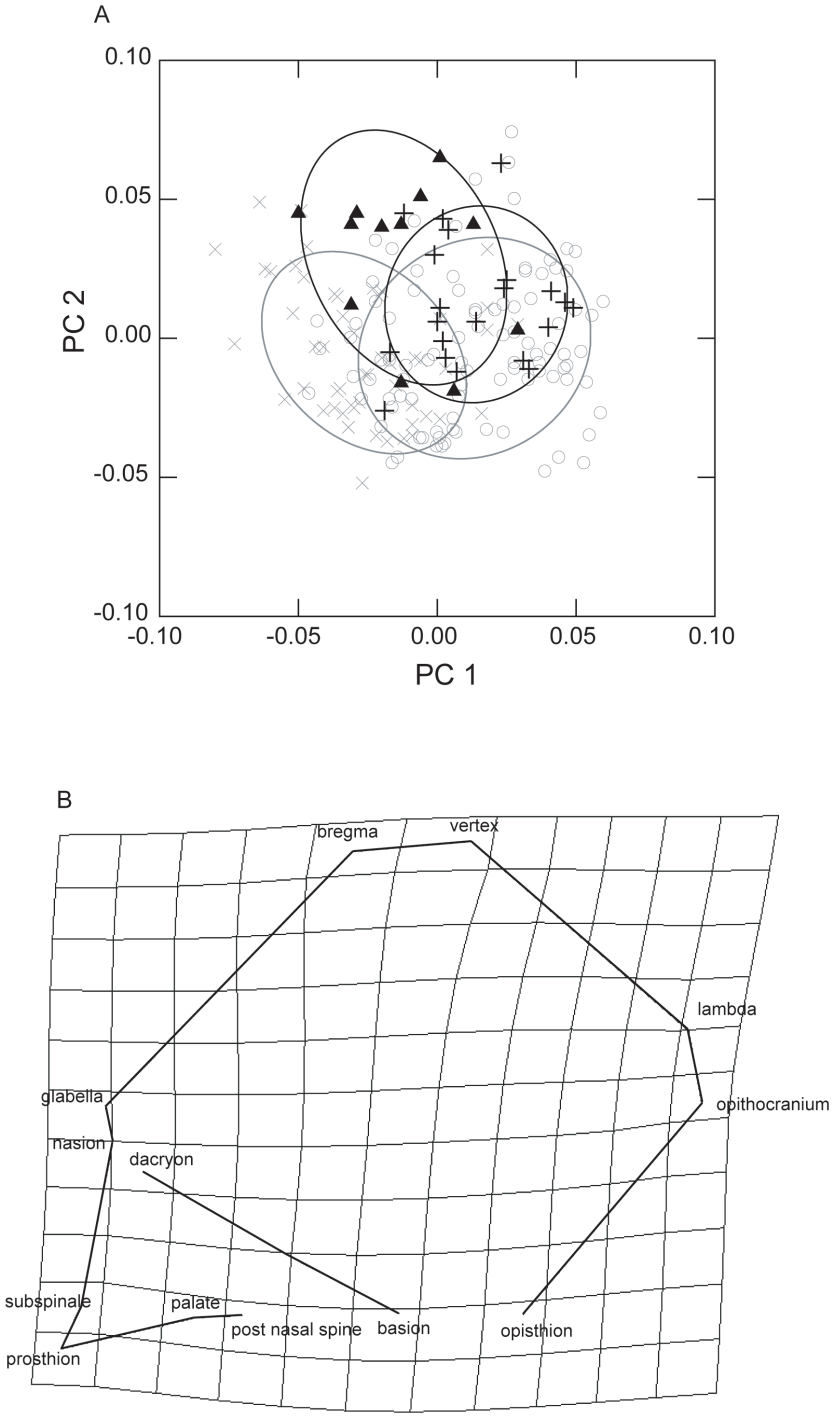
Differentiation according with PCA. (A) Samples distribution according with PC1 vs. PC2. Ellipses represent the 95% confidence interval of distributions for each sample. X: Eastern non-pygmies; O: Western non-pygmies; **Δ**: Eastern pygmies; +: Western pygmies. B) Transformation grids on PC1 in lateral view. Eastern groups were considered the reference and western groups, the target. Basilar and frontal views do not represent any important variation.

The first 31 PCs explain 90% of shape variation. Significant differences between groups in the first five PCs are present in table 1. Main differentiation represented by PC1, which explains 16.83% of variation, concerns Eastern and Western groups (p<0.01) (Table 1, Fig. 1A). The PC2, which has an eigenvalue (12.2%) almost as large as the first PC, and PC3 differentiate mainly Pygmies from non-pygmies (Table 1, Fig. 1B). The following PCs separate Eastern non-pygmies (PC4) and Western non-pygmies (PC5) from the other groups. The overlapping of different groups, according with PCs 1 and 2 (Fig. 1A), would indicate most of shape variance is among crania rather than among groups; however, such overlapping is not uncommon when populations geographically and genetically related are compared.

**Table 1:** Percentage of variation explained by first five PCs, ANOVA and post hoc LSD to test differences between groups.

	eigenvalue	% of variation	F	p	pairwise LSD comparison
CS			2.87	0.038	ENp≠WP*; WNp≠WP*
PC1	0.00096	16.8	31.36	0.000	ENp≠WNp**; ENp≠WP**; WNp≠EP**; EP≠WP**
PC2	0.0007	12.2	8.52	0.000	ENp≠EP**; ENp≠WP**; WNp≠EP**; WNp≠WP*
PC3	0.00046	8.07	9.02	0.000	ENp≠EP**; ENp≠WP**; WNp≠EP**; WNp≠WP*
PC4	0.00039	6.87	8.14	0.000	ENp≠WNp**; ENp≠EP*; ENp≠WP**; WNp≠WP*
PC5	0.0003	5.23	11.73	0.000	ENp≠WNp**; WNp≠EP**; WNp≠WP**

ENp: Eastern non-pygmies; WNp: Western non-pygmies; EP: Eastern pygmies; WP: Western pygmies.

*: p<0,05;

**: p<0,01.

All Mahalanobis distances obtained from the 31 PCs are highly significant (p<0.01) (Table 2). The greatest morphologic distances occur between Eastern non-pygmies and pygmies, followed by distances of both pygmies groups with respect to Western non-pygmies. The shortest distance is obtained between Eastern and Western non-pygmies.

**Table 2:** Mahalanobis distances between groups adjusted using van Vark's method.

	Eastern Np	Western Np	Eastern P
Western Np	8.742		
Eastern P	15.806	11.416	
Western P	16.749	11.85	10.728

**Figure 2:**
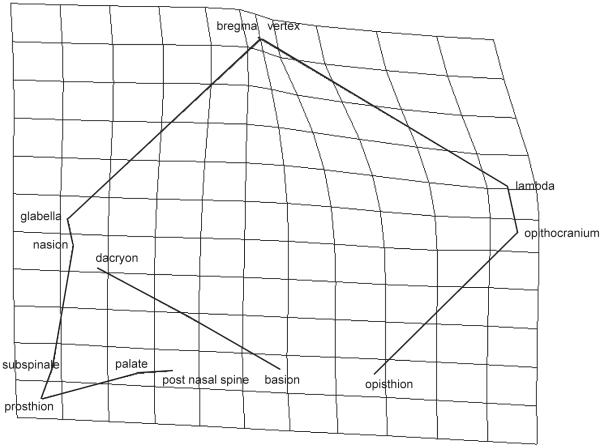
Transformation grids on PC2 in lateral (A) view. Non-pygmies were considered the reference and Pygmies, the target. Frontal and basilar views do not represent any important variation.

Shape variation indicates that western groups present longer vaults and shorter faces than eastern groups (Fig. 1B). Variation on PC2 involves differentiation in the shape produced mainly by the short distance between bregma and vertex (Fig. 2) being the vertex more anteriorly located in pygmies than in non-pygmies.

**Figure 3:**
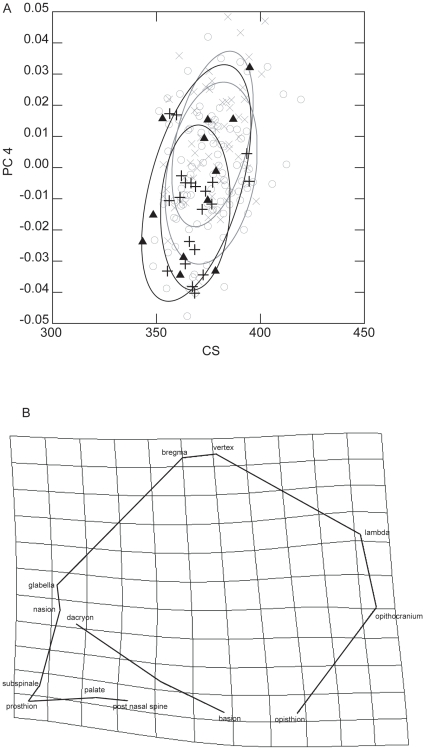
Differentiation explained by allometries and between-groups variation. (A) Samples distribution according with CS vs. PC4. Ellipses represent the 95% confidence interval of distributions for each sample. X: Eastern non-pygmies; O: Western non-pygmies; **Δ**: Eastern pygmies; +: Western pygmies. (B) Transformation grids on PC4 in lateral (left) view. Eastern non-pygmies were considered the reference and Western pygmies, the target. Frontal and basal views do not represent any important variation.

**Figure 4:**
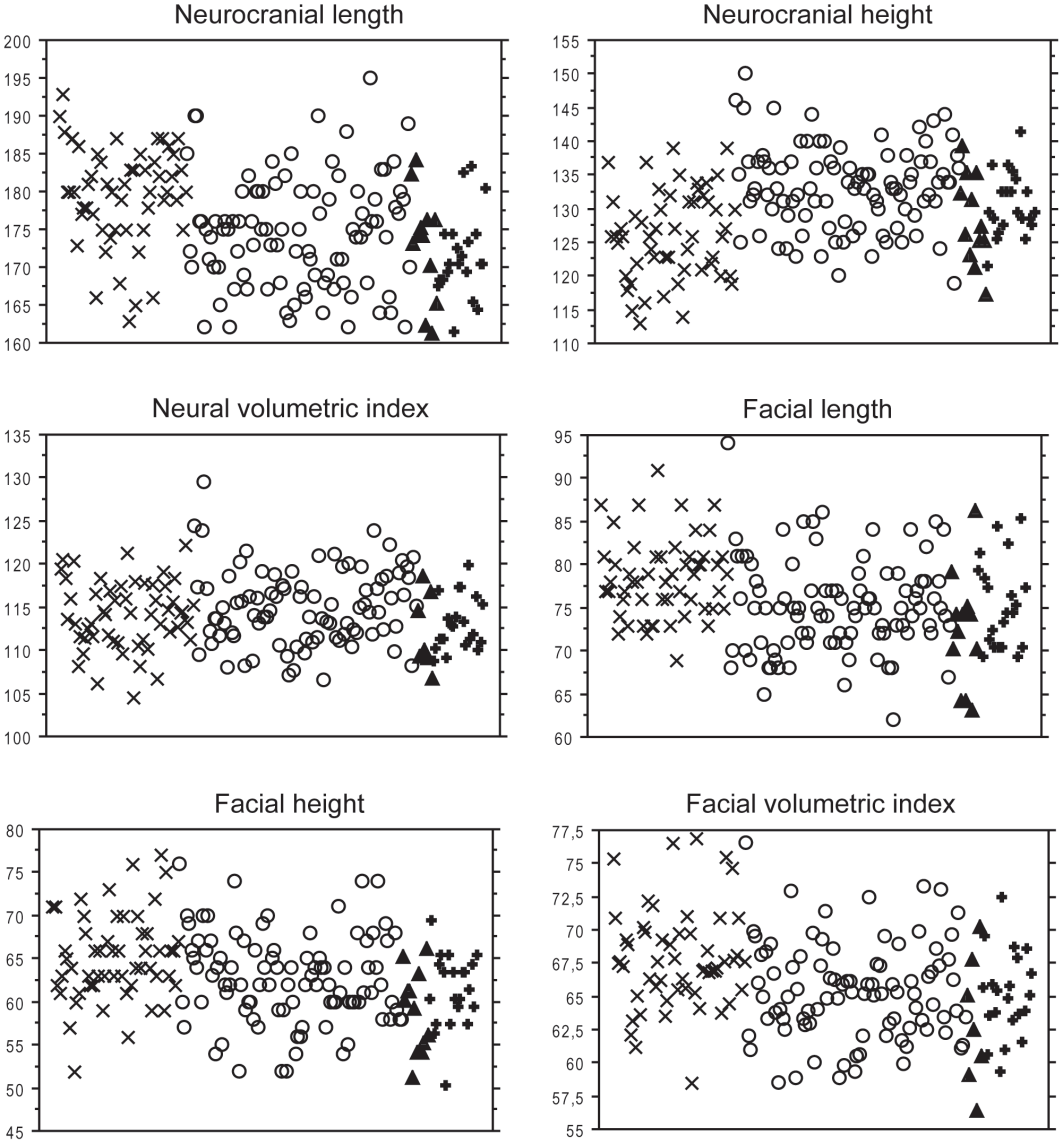
Values distribution for major cranial components. X: Eastern non-pygmies; O: Western non-pygmies; **Δ**: Eastern pygmies; +: Western pygmies. Minimum and maximum values are respectively as follows: Neurocranial length (163–193) (162–195) (161–184) (161–183), Neurocranial height (113–139) (119–150) (117–139) (121–141), Neural volumetric index (105–122) (107–130) (107–118) (108–119), Facial length (69–91) (62–94) (63–86) (69–85), Facial height (52–77) (52–76) (51–66) (50–69), Facial volumetric index (58–77) (58–77) (56–70) (59–72).

Differences in CS were significant (p<0.05) and they concerns Western pygmies which show a smaller cranial size than both Eastern and Western non-pygmies; it is worth to note that Eastern pygmies do not show significant difference in size with any group (Table 1, Fig 3A). When measurements are considered (Fig 4), the neural volumetric index does not show significant difference between groups; dissimilarities are observed between Eastern non-pygmies and the others groups in the neural length and height, the same can be said between Western pygmies and Western non-pygmies. Difference between Eastern non-pygmies and the others groups are also observed in the facial volumetric index as well as in facial length and height (Table 3).

**Table 3:** ANOVA and post hoc LSD to test differences between groups.

variable	F	p	difference between groups
*major cranial components* °			
NL	10.58	0.000	ENp>WNp **; ENp>EP**; ENp>WP**; WNp>WP*
NW	1.29	0.278	
NH	14.96	0.000	ENp>WNp**; ENp>WP*; WNp>WP*
NVI	2.3	0.078	
FL	7.63	0.000	ENp>WNp**; ENp>EP**; ENp>WP**
FW	2.27	0.081	
FH	7.61	0.000	ENp>WNp**; ENp>EP**; ENp>WP**; WNp>EP*
FVI	8.1	0.000	ENp>WNp**; ENp>EP**; ENp>WP**

°see Table 7 for explanation.

### Allometries

The multivariate regression was highly significant (Wilk's λ = 0.525; F = 4.379; p<0.000) and explained 1.62% of variance of 31 PCs. PCs with a slope associated with CS are: 4, 17, 26 and 30 (p<0.01) and 20 and 28 (p<0.05) (Table S1). Significant shape variation associated to size variation related between groups (ANOVA, post hoc LSD) is only represented by PC4 (Table 4). PC4 explains only 6.87% of the total variance derived from the GPA/PCA (Table 1) and is mainly related to the distinction between Eastern non-pygmies from the other groups. Samples distribution of CS against PC4 is plotted in figure 3A. Western pygmies appear as showing smaller size than non-pygmies whereas Eastern Pygmies are widely distributed. Transformation grid (Fig. 3B) indicates that Western pygmies present smaller face and a little shorter and higher neurocranium than both non-pygmies.

**Table 4:** ANOVA and post hoc LSD of PCs associated with CS.

PC	F	p
4	8.14	<0.01
17	1.58	0.19
20	0.43	0.73
26	1.79	0.15
28	0.49	0.69
30	1.04	0.37

## Discussion

Cranial shape may have a strong genetic component and morphologic distances would represent genetic distances [19]. Plasticity and environment are not supposed to affect size and shape variation since only populations inhabiting tropical rainforest were included in this work. Since African pygmies share a most recent common ancestor [13]–[16] and are living in similar environmental conditions, it is expected that cranial shape and size between pygmies are closer between them than to any other non-pygmy population (first hypothesis). The sample size in our study is not homogenous, being Eastern pygmies less represented. However, all African pygmies available for study were included.

Results of the PCA indicate that the main cranial shape differentiation does not occur between pygmies and non-pygmies but between Eastern and Western populations (pygmies and non-pygmies) (Table 1; Fig. 1A); all comparison between East and West groups reach a high level of significance (p<0.01). Eastern non-pygmies appear as the most distinctive group and differentiate from pygmies and Western non-pygmies in relative and absolute values. Since western pygmies show a higher level of admixture than eastern pygmies, it can be expected that the former differentiate less than the latter from non-pygmies. However, a reduction of cranial size is observed in Western pygmies with a lower level of significance (p<0.05). This character is not shared by Eastern pygmies who show a great variation (observed already by Thilmans [23]) overlapping with all other groups. Distinction between pygmies and non-pygmies is provided by PC2 and PC3 which explains 12.2% and 8.07% of variation. In these PCs, high level of significance (p<0.01) is observed between pygmies and non-pygmies suggesting a clear distinction between them, although it appears as less evident than the differentiation between East and West groups (PC1).

According to multivariate (Mahalanobis) and univariate analyses (Tables 2 and 3), distance between both pygmy groups, although highly significant, was shorter than the distance between pygmies and non-pygmies but higher than the distance between non-pygmies. Thilmans [23] in his analysis of Mahalanobis distances based on ten measurements of eleven series of skull observed that shorter distance occur between Eastern and Western pygmies but also between Eastern pygmies and non-pygmies groups from Gabon and African Central Rep. These results suggest that African pygmies groups share a more recent common ancestor than with non-pygmies, observation which agrees with data from genetic works [13]–[16]. East and West non-pygmies show the shorter distance arguing also thus for a recent common ancestor. Higher distances concern East non-pygmies when compared with pygmies followed by the distance between pygmies and West non-pygmies. The distinction between pygmies and non-pygmies is evident and the first hypothesis cannot be rejected. However, the high differentiation between Eastern and Western groups, both pygmies and non-pygmies, cannot be disregarded.

Overall cranial size variation between groups was lower than shape variation (Table 1). Since the second hypothesis states that pygmies show smaller craniofacial size than non-pygmies populations, it would be expected that both Eastern and Western pygmies present significant differences in CS in relation to both non-pygmies groups and that measurements in pygmies are smaller than in non-pygmies. The CS variation concerns only Western pygmies in relation to non-pygmies (Table 1). Variation in measurements and indices can give more insight about size. Western pygmies are smaller in neural dimensions than non-pygmies but they differentiate only from Eastern non-pygmies in facial dimensions (Table 3). Size difference in Eastern pygmies involves few aspects (i.e. Neural length and Facial dimensions) and only in relation to Eastern non-pygmies. In sum, measurements suggest that craniofacial size in pygmies appears smaller than in Eastern non-pygmies, but whereas Western pygmies differentiate also from Western non-pygmies, Eastern pygmies cannot be distinguished from these. Thus, whereas CS does not show size difference between Eastern pygmies and non-pygmies, linear measurements suggest that Eastern pygmies present a smaller skull than Eastern non-pygmies. Both methods fail to find differences between Eastern pygmies and Western non-pygmies. Thus, pygmies do not present systematically low values in craniofacial size; it suggests that variation in pygmies overlaps completely or in very high proportion with the variation of non-pygmies (Fig 4). Having in mind that the second hypothesis states that pygmies show smaller craniofacial size than non-pygmies populations, the presence of clear size reduction only in Western pygmies leads to reject the hypothesis. However, if Western and Eastern pygmies are considered separately, this second hypothesis is rejected for Eastern pygmies but it is not for Western pygmies.

Size variation was associated with changes in shape; however, the axis of maximal shape variation (PC1) was not the most correlated with centroid size. The PC4 is the only PC which correlates with centroid size, which is associated with significant differentiation between groups. Size-related shape variation involves a smaller face and shorter neurocranium in Western pygmies in relation to non-pygmies. However, Eastern pygmies do not show a clear pattern in the distribution (Fig. 2). Since the hypothesis states a particular cranial shape is associated with size differentiation in pygmies, the lack of difference between Easter pygmies and Western non-pygmies suggest that the hypothesis has to be rejected. However, if pygmies groups are considered separately, the hypothesis is rejected for Eastern pygmies but not for Western pygmies. This distinction reinforces the suggestion that African pygmies do not behave as a homogeneous group.

Allometry in body proportion and between body size and tooth size has been evaluated in African pygmies [30]–[31]. If allometry occurs in cranial morphology, cranial size and shape of pygmies should be similar to those of sub-adult non-pygmies. In this work, only adult individuals were studied and thus this hypothesis cannot be directly tested. However, our results suggest that the smallest cranial size of Western pygmies, although associated with shape variation, was not associated with the main size variation among African rain forest groups.

Each cranial component is characterised by a distinctive growth pattern and shows some level of integration with respect to other components [44]. Cranial components exhibit their own maturity gradient [45]. The neurocranium attains adult size and shape early in development with a clear reduction in rate of growth around 2.5 years of age [46]. In contrast, the face undergoes substantial changes until to the adult stage, being vertical measurements of maxilla and mandible the most retarded in development [44], [47], [48]. According to the cranial maturity gradient, it would be expected that the less mature structures are more affected by a given factor than the more mature structures [49]. When pygmies are compared with non-pygmies, differences in Eastern pygmies are not the same as those of Western pygmies. Indeed, facial height is significantly smaller in Eastern pygmies in relation to non-pygmies' groups, whereas Western pygmies are distinguished from non-pygmies by showing smaller neural dimensions (Table 3). Following the criterion of Buschang and Hinton [49], and assuming that there are common factors affecting cranial and post-cranial morphology, it seems as if the factor responsible for short adult size in Eastern pygmies acts once neural development has attained an advanced degree of development. It means that the attainment of a brain size similar to that of non-pygmies would result from truncation of growth trajectories in Pygmies in the juvenile stage (around 7–11 years old). This would occur when brain size is very close to the adult size, but facial structures have not yet reached adult size. However, this hypothesis does not fit with the data from longitudinal studies in new born and infants pygmies. Bailey [50] reported that at birth Efe pygmies (Eastern pygmies) show lower weight and reduced size than the Lese (their non-pygmies neighbours) and that the differences persist over the next 5 years. However, van Eijk [51] found that reduction in size in Baka (Western pygmies) does not start before the age of 4 years. It appears that developmental mechanisms leading to morphological differentiation may differ among pygmies.

It has been suggested that the short stature of pygmies is the result of a deficiency in the GH axis. Even if cranial structures are not the best place to observe the influence of such deficiency, several studies have indicated that a reduction in GH/IGF 1 produce many modifications in cranial size and shape: maxilla, mandible and sphenoid bone are shorter, orbits are shallow, and head circumference is smaller [52]–[55]. In a global GH-IGF1 deficiency scenario, one can expect that all or almost all measurements in pygmies are smaller than in non-pygmies. This pattern does not fit well with the overall morphological differentiation obtained with our results.

We have tested in this work if African pygmies appear as a homogenous group with respect to non-pygmies living in tropical rainforest. If African pygmies appear as a homogenous group it would justified to pool African pygmies together in works on cranial morphology. In order to gain some insight on pygmy biology, we have also tested allometries. Comparison of cranial morphology between pygmies and non-pygmies groups shows that important differences in shape concern Western vs Eastern populations (pygmies and non-pygmies) and that a smaller size in skull is only observed in Western pygmies, whereas Eastern pygmies overlap with all other groups. Shape changes associated to size modifications are very low and allometric changes seem to affect only Western pygmies. Therefore, our findings raise serious doubt about the fact to consider African pygmies as a homogenous group in studies on skull morphology.

Although our results agree with suggestions that African pygmies share a more recent ancestor [13]–[15], differences in cranial morphology suggest a post-split independent skull differentiation. There is no direct evidence to associate independent skull differentiation with independent body size reduction, but we cannot disregard the possibility that the process of pygmeisation occurred after the split of the ancestors of modern Eastern and Western pygmies. Patin et al. [14] suggested that the split of pygmy's ancestor into Easter and Western populations occurs around 20,000 years ago. Our knowledge of this original population and the habitat in which they lived is very limited [56] but some clues may be found in studying climatic changes. OIS 2 represents a cooling event which starts around 24,000 years ago and finished around 12,000 years ago. Global temperature minima are associated with reduced rainfall in tropical Africa with subsequent reduction in the areas occupied by the rainforest [57]–[59]. Such decrease of the rainforest certainly happened during OIS 2. The global warming that followed led to the replacement of open, grassy vegetation by rain forest and by about 8,000 years ago, rainforest reached maximum extension [see 60]. Patin et al. [14] have suggested that the split between Eastern and Western pygmies occurred at the same time as the rainforest retreated into refugia. However, they observed that gene flow has continued and only stopped more recently. However, one can also hypothesize that around 20.000 years ago, a population living around the periphery of the rain forest, moved to the East and West following the shrinkage of the forest and the expansion of the savanna, which at this point was nearing the Equator as a consequence of climatic changes associated with global cooling event (OIS 2). With warmer conditions at the end of the Pleistocene, the rain forest extended, and due to demographic pressures, populations inhabiting the periphery cannot move to the north and have to adapt to live in the rain forest. Gene flow between Eastern and Western populations stops at that moment (Bagyeli and Baka pygmies who inhabit in South Cameroon, in territories separated by few hundred kilometers, were never in contact and did not know the existence of each other until few years ago). The process of pygmeisation started at that time, in this new habitat, but it developed differently in Eastern and Western regions. Such scenario agrees with the archeological evidence which points to the first presence of settlements in the forest at the end of Pleistocene [61] and it would explain why a) gene flow between Eastern and Western pygmies' ancestors does not stop around 20,000 years but later, b) why pygmies do not appear as a homogenous group in relation to non-pygmies populations, probably also due to a different degree of gene flow with non-pygmies groups [14], and c) why Eastern and Western pygmies differ in cranial and skeletal morphology [24], [28, this study]. This scenario also predicts that potential biological mechanisms that produce differentiation in pygmies (e.i. GH/IGF) would not be the same for all African pygmies.

## Materials and Methods

Four cranial samples composed of 182 individuals were analyzed (Table 5). Individuals were grouped into Western pygmies (Gabon, Congo, Central-African Rep.), Eastern pygmies (Democratic Rep. of Congo), Western non-pygmies (Gabon, Congo, Cameroon, Central-African Rep., Democratic Rep. of Congo) and Eastern non-pygmies (Democratic Rep. of Congo, Rwanda) and are housed at the Musée de l'Homme (Paris), Institut de Paléontologie Humaine (Paris), Institut Royal des Sciences Naturelles de Belgique (Brussels), British Museum of Natural History (London) and University of Geneva. Marquer [27], Adé [62], Bakonyi [63] and Thilmans [23]–[24] provided detailed information about geographical provenience and historic events for each skull attributed to pygmies. Pygmies are represented by a small sample size but they correspond to all skulls available around the world for study [see 23]. Both non-pygmy samples include only groups that inhabit the tropical rainforest and/or in contact with pygmies. Terms referring to these populations as they figure in museums' records are Adouma, Ashango, Banghi, Bondjo, Bopan, Boupara, Bwiti, Hutu, Itsogho, Kalai, Kale, Luba, Mbagha, Mbenga, Mpongue, Pahouin, Sendi, Teke, Vanyaneka, Wangi, Yakoma, Yanda, Yaka. Some Pygmies were sexed by Marquer [27] based on postcranial bones; others Pygmies and Non-pygmies were sexed, when possible, using classical standards [64]. Sexes were pooled together in statistical analyses.

**Table 5:** Sample composition.

	Eastern Np	Western Np	Eastern Pygmies	Western Pygmies
males	27	48	4	5
females	27	42	3	8
unknown	0	5	5	8
**total**	**54**	**95**	**12**	**21**

Thirty three-dimensional (3D) landmarks, located in the vault, basicranium, and face were registered with Microscribe on the left side of the skull (Table 6). Cranial variation was firstly assessed multivariately. Tri-dimensional configurations of 29 landmarks were subject to Generalized Procrustes analysis (GPA) and scaled according with the centroid size (CS) (the square root of the sum of square distances from each landmark to the specimen's centroid) [65]–[67]. Shape was defined as the residual geometric information remaining once differences due to location, scale, and rotational effects were removed [65]–[66]. After Procrustes transformation, the coordinates were projected in the tangent space to Kendall's shape space and the resulting shape information was subject to Principal Components Analysis (PCA) [67]–[69]. The morphometric analyses were performed in Morphologika [67]. The principal components (PCs) of tangent coordinates represent axes of maximal shape variation. Only those PCs whose eigenvalues sum at least 90% of the variation were retained for further analysis.

**Table 6:** Landmarks and definitions.

Glabella	1	Most anterior point of the frontal bone at the sagittal plane
Bregma	2	Intersection of coronal and sagittal sutures
Vertex	3	Most superior point of the vault at the sagittal plane
Lambda	4	Intersection of the sagittal and lambdoidal sutures
Opisthocranium	5	Most posterior point of the skull at the sagittal plane
Opisthion	6	Midline point on the posterior margin of the foramen magnum
Basion	7	Midline point on the anterior margin of the foramen magnum
Hormion	8	Most posterior midline point of the vomer
Pterion	9	Region where the frontal, parietal, sphenoid and temporal joint
Euryon	10	Most lateral point of the vault at the parietal bone
Asterion	11	Intersection of lamboidal, perimastoid and occipitomastoid sutures
Lesser wing of the sphenoid	12	Midpoint of the septum between the optic foramen and the superior orbital fissure
Dacryon	13	Point where the lacrimomaxillary suture joins the frontal bone
Ectoconchion	14	Most lateral point of the orbital margin
Supraorbitary	15	Most superior point of the orbital margin
Orbitale	16	Most inferior point of the orbital margin
Nasion	17	Intersection of internasal and frontonasal sutures
Subspinale	18	Deepest point of the subspinale concavity
Posterior nasal spine	19	Most posterior point of palatal bones
Right alare	20	Most lateral point of the right side of the nasal aperture
Left alare	21	Most lateral point of the left side of the nasal aperture
Zygomaxillare	22	Lowest point of the zygomaticomaxillary suture
Inferior zygotemporal	23	Lowest point of the zygotemporal suture
Glenoid	24	Most posterior point of the glenoid fossa
Sphenotemporal	25	Most external point of the sulcus located forward of the sphenotemporal crest
Stephanion	26	Intersection between the coronal suture and the inferior temporal line
Prosthion	27	Most anterior point of the alveolar processes of the maxillae
Posterior alveolar	28	Most posterior limit of the maxillary alveolar arch
Palate	29	Intersection of the palatine and maxillary bones

Landmarks were also used to obtain linear measurements and volumes as complementary comparison of size between groups. The skull was divided into two major components, face and neurocranium, and three orthogonal distances were measured for each one, length, width and height (Table 7). Volumetric indices representing the geometric mean of the three dimensions were constructed to estimate size variation [70]–[72].

**Table 7:** Measurements and indices.

Abbreviation	
*major cranial components*	
NL	Neurocranial length: Nasion-Opistocranium
NW	Neurocranial width: Eurion-Vertex lateral projection
NH	Neurocranial height: Basion-Vertex
NVI	Neural volumetric index: geometric mean between NL, NW and NH
FL	Facial length: Prosthion-Hormion
FW	Facial width: Prosthion-Zygion lateral projection
FH	Facial height: Nasion-Prosthion
FVI	Facial volumetric index: geometric mean between FL, FW and FH

Statistical analyses encompasses: A) Discriminant Analysis was performed with PCs to obtain Mahalanobis distances between groups in order to test the first hypothesis. Discriminant analysis was not performed with the scores after Procustes transformation because they represent a high quantity of variables, 87 (29 landmarks×3D), to discriminate among 182 individuals, mainly considering that pygmy groups have small sample sizes. PCs instead retain much part of variation, 90%, with a lesser number of variables. Because both pygmy groups have a small sample size, Mahalanobis distances, D^2^, were adjusted using the method proposed by van Vark et al. [73], calculating an unbiased Mahalanobis distance, Δ^2^, as follows:
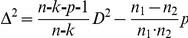
where n is the total sample size, k is the number of samples being compared, n_1_ and n_2_ are the sizes of the samples whose D^2^ value is calculated, p is the number of variables [73: 403]. B) Multivariate regression of the shape variables (the principal components, PCs) on CS was performed to assess allometries, for testing the third hypothesis. C) Univariate differentiation between groups in CS, main PCs, measurements, and volumetric indices were evaluated with ANOVA and post hoc LSD, in order to test the second and the third hypotheses.

## Supporting Information

Table S1 Multivariate regression results.
(0.07 MB DOC)
