# Supplementary material for: Diversity among African Pygmies
Source: PLoS One. 2010 Oct 26;5(10):e13620. doi: 10.1371/journal.pone.0013620 (PMC2964320; doi:10.1371/journal.pone.0013620)
Supplement: Table S1 — Multivariate regression results. (0.07 MB DOC) [file pone.0013620.s001.doc]

| **Table S1. Multivariate regression results** | | | | |  |
| --- | --- | --- | --- | --- | --- |
| PC |  | Coefficient | Std error | t-stat | p |
| 1 | Intercept | -0.079 | 0.062 | -1.269 | 0.206 |
|  | slope | 0.000 | 0.000 | 1.270 | 0.206 |
| 2 | Intercept | 0.026 | 0.053 | 0.486 | 0.627 |
|  | slope | 0.000 | 0.000 | -0.487 | 0.627 |
| 3 | Intercept | -0.039 | 0.043 | -0.899 | 0.370 |
|  | slope | 0.000 | 0.000 | 0.900 | 0.369 |
| 4 | Intercept | -0.184 | 0.038 | -4.899 | 0.000 |
|  | slope | 0.000 | 0.000 | 4.902 | 0.000 |
| 5 | Intercept | -0.061 | 0.035 | -1.772 | 0.078 |
|  | slope | 0.000 | 0.000 | 1.773 | 0.078 |
| 6 | Intercept | -0.040 | 0.034 | -1.170 | 0.244 |
|  | slope | 0.000 | 0.000 | 1.171 | 0.243 |
| 7 | Intercept | 0.021 | 0.029 | 0.717 | 0.475 |
|  | slope | 0.000 | 0.000 | -0.717 | 0.474 |
| 8 | Intercept | -0.003 | 0.026 | -0.095 | 0.924 |
|  | slope | 0.000 | 0.000 | 0.096 | 0.924 |
| 9 | Intercept | -0.002 | 0.025 | -0.071 | 0.944 |
|  | slope | 0.000 | 0.000 | 0.071 | 0.944 |
| 10 | Intercept | 0.007 | 0.025 | 0.289 | 0.773 |
|  | slope | 0.000 | 0.000 | -0.289 | 0.773 |
| 11 | Intercept | 0.034 | 0.022 | 1.535 | 0.127 |
|  | slope | 0.000 | 0.000 | -1.536 | 0.126 |
| 12 | Intercept | 0.001 | 0.022 | 0.067 | 0.946 |
|  | slope | 0.000 | 0.000 | -0.068 | 0.946 |
| 13 | Intercept | 0.029 | 0.020 | 1.399 | 0.164 |
|  | slope | 0.000 | 0.000 | -1.399 | 0.163 |
| 14 | Intercept | -0.011 | 0.020 | -0.545 | 0.587 |
|  | slope | 0.000 | 0.000 | 0.545 | 0.586 |
| 15 | Intercept | -0.018 | 0.019 | -0.966 | 0.336 |
|  | slope | 0.000 | 0.000 | 0.966 | 0.335 |
| 16 | Intercept | 0.016 | 0.018 | 0.892 | 0.374 |
|  | slope | 0.000 | 0.000 | -0.893 | 0.373 |
| 17 | Intercept | 0.051 | 0.018 | 2.936 | 0.004 |
|  | slope | 0.000 | 0.000 | -2.938 | 0.004 |
| 18 | Intercept | 0.004 | 0.017 | 0.234 | 0.815 |
|  | slope | 0.000 | 0.000 | -0.235 | 0.815 |
| 19 | Intercept | 0.006 | 0.017 | 0.359 | 0.720 |
|  | slope | 0.000 | 0.000 | -0.359 | 0.720 |
| 20 | Intercept | -0.034 | 0.016 | -2.124 | 0.035 |
|  | slope | 0.000 | 0.000 | 2.126 | 0.035 |
| 21 | Intercept | -0.027 | 0.015 | -1.740 | 0.084 |
|  | slope | 0.000 | 0.000 | 1.741 | 0.083 |
| 22 | Intercept | -0.027 | 0.015 | -1.815 | 0.071 |
|  | slope | 0.000 | 0.000 | 1.816 | 0.071 |
| 23 | Intercept | 0.013 | 0.014 | 0.923 | 0.357 |
|  | slope | 0.000 | 0.000 | -0.924 | 0.357 |
| 24 | Intercept | 0.009 | 0.014 | 0.664 | 0.508 |
|  | slope | 0.000 | 0.000 | -0.664 | 0.507 |
| 25 | Intercept | -0.012 | 0.013 | -0.928 | 0.355 |
|  | slope | 0.000 | 0.000 | 0.929 | 0.354 |
| 26 | Intercept | 0.041 | 0.013 | 3.158 | 0.002 |
|  | slope | 0.000 | 0.000 | -3.160 | 0.002 |
| 27 | Intercept | 0.023 | 0.013 | 1.825 | 0.070 |
|  | slope | 0.000 | 0.000 | -1.826 | 0.070 |
| 28 | Intercept | -0.026 | 0.012 | -2.119 | 0.035 |
|  | slope | 0.000 | 0.000 | 2.120 | 0.035 |
| 29 | Intercept | 0.014 | 0.012 | 1.166 | 0.245 |
|  | slope | 0.000 | 0.000 | -1.167 | 0.245 |
| 30 | Intercept | 0.035 | 0.011 | 3.056 | 0.003 |
|  | slope | 0.000 | 0.000 | -3.058 | 0.003 |
| 31 | Intercept | -0.015 | 0.011 | -1.333 | 0.184 |
|  | slope | 0.000 | 0.000 | 1.334 | 0.184 |
